# Differential Effectiveness of Aromatase Inhibitors in Hormone Receptor–Positive Breast Cancer

**DOI:** 10.1001/jamanetworkopen.2025.51548

**Published:** 2025-12-01

**Authors:** Laura C. Pinheiro, Shoshana M. Rosenberg

**Affiliations:** Division of General Internal Medicine, Department of Medicine, Weill Cornell Medicine, New York, New York (Pinheiro); Division of Epidemiology, Department of Population Health Sciences, Weill Cornell Medicine, New York, New York (Pinheiro, Rosenberg); Cancer Prevention and Control Research Program, Sandra and Edward Meyer Cancer Center, Weill Cornell Medicine, New York, New York (Pinheiro, Rosenberg).

Adjuvant endocrine therapy (ET) for individuals with hormone receptor–positive (HR^+^) breast cancer is recommended for at least 5 and up to 10 years after primary breast cancer treatments end.^1^ Suboptimal ET adherence and early ET discontinuation are well-documented challenges that contribute to poorer HR^+^ breast cancer outcomes. ET adherence and early discontinuation rates vary across studies, with estimates reported in 1 systematic review to range from 41% to 72% and 31% to 73% by 5 years, respectively.^2^ While reasons for nonadherence and discontinuation are multifaceted,^3^ data reflecting ET use and outcomes outside of a clinical trial setting can better capture real-world patterns, potentially informing targets for intervention to improve ET adherence and persistence and, ultimately, survival. Dumas and colleagues^4^ sought to generate such evidence by leveraging randomized clinical trial emulation methods to study nearly 150 000 women aged 50 to 75 years in France with early-stage breast cancer who initiated adjuvant aromatase inhibitor (AI) therapy without ovarian suppression. With a median follow-up of 63 months, Dumas et al^4^ observed the lowest 8-year disease-free survival (DFS) and overall survival (OS) for individuals taking exemestane compared with anastrozole and letrozole. Although discontinuation was highest among the exemestane group, lower DFS and OS were observed even assuming perfect persistence. This finding offers an additional data point for medical oncologists to consider as they make adjuvant treatment recommendations for postmenopausal women with breast cancer.

The study by Dumas and colleagues^4^ deepens our understanding regarding the current adjuvant ET landscape for postmenopausal women with breast cancer. First, it sheds light on salient clinical outcomes—DFS and OS—in a clinical setting, which is likely more generalizable than results generated in a randomized clinical trial population.^5^ Although the investigators used inverse probability weighting to adjust for several demographic and clinical factors, findings from the current study may reflect potential (unmeasured) confounding by indication (ie, patient, clinician, or disease characteristics that may drive prescription of exemestane and also be associated with survival). Second, the large sample size in this study of nearly 150 000 women across France is an important strength, especially because the original clinical trials may have been underpowered to detect small differences. At the same time, given the large sample size, small between-group differences that are statistically significant may not be clinically meaningful. The modest observed differences in survival underscores the importance of considering other outcomes, such as adverse effect burden and health-related quality of life (HRQOL), which are known to be negatively impacted by ET.^6^ The current study did not include HRQOL or any other patient-reported outcomes but did report minimal differences in adverse events, which they considered incident dyslipidemia, diabetes, and bone fractures. Accounting for HRQOL and other patient-reported measures into observational studies would substantially strengthen their impact for providing evidence to support shared decision-making between patients and their oncologists.

While the current analysis spans a 10-year period, the cohort is limited to women diagnosed through 2020, which may not reflect more contemporary treatment regimens; given the emergence of newer targeted therapies (ie, cyclin-dependent kinase [CDK]4 inhibitors such as ribociclib) that may be prescribed in the setting of high-risk, early-stage HR^+^ breast cancer in combination with an AI such as letrozole or anastrozole.^7^ Future analyses using population-based data inclusive of these newer treatments should further inform their impact on adherence, persistence, and clinical outcomes in an empirical setting.
